# Examining Predictors of Psychiatric Hospitalization Outcomes in Older Adults With and Without Dementia

**DOI:** 10.1093/geroni/igaf122.3762

**Published:** 2025-12-31

**Authors:** Sophia McInturff, Cindy Woolverton, Rafael Samper-Ternent, Carolyn Pickering, Michelle Patriquin, Scott Lane

**Affiliations:** University of Texas HSC at Houston, Houston, Texas, United States; UTHealth Houston, Houston, Texas, United States; University of Texas Health Science Center at Houston, Houston, Texas, United States; UTHealth, Cizik School of Nursing, Houston, Texas, United States; University of Texas HSC at Houston, Houston, Texas, United States; University of Texas HSC at Houston, Houston, Texas, United States

## Abstract

Older adults with dementia present for acute inpatient psychiatric care to manage dementia-related neuropsychiatric symptoms and co-occurring mental health conditions. Given the complexity of needs, these individuals may be at increased risk for poor outcomes when compared to those without dementia, such as prolonged hospitalization and fragmented discharge planning. This study aimed to examine predictors of health outcomes among this population. Electronic health record data was extracted for patients aged 65+ admitted to the UTHealth Houston Behavioral Sciences Center from 2021-2024. Regression models assessed predictors of length of stay (LOS) and admission type (involuntary vs. voluntary) for individuals with (n = 94) and without (n = 276) a dementia diagnosis. Results indicated that dementia diagnosis, financial status (payer status: indigent, government-assisted, or secure), psychotic disorder diagnoses, and discharge disposition significantly predicted LOS. The length of stay increased by an estimated 3 days with a dementia diagnosis, 3 days with a psychotic disorder, and 20 days with secure payer status. Discharge to assisted living was more common among individuals with dementia (41.7%) than those without (13.9%). The logistic regression predicting admission type was significant (χ²(4) = 32.23, p < .001); dementia diagnosis, psychosis symptoms, and secure payer status increased the likelihood of involuntary admission. These findings suggest that older adults with dementia are at increased risk for longer hospitalizations and involuntary admissions, highlighting the need for tailored discharge planning and improved care transitions in psychiatric settings.

